# Significant improvement after training in the assessment of lateral compartments and short-axis measurements of lateral lymph nodes in rectal cancer

**DOI:** 10.1007/s00330-022-08968-0

**Published:** 2022-07-08

**Authors:** Tania C. Sluckin, Sanne-Marije J. A. Hazen, Karin Horsthuis, Doenja M. J. Lambregts, Regina G. H. Beets-Tan, Pieter J. Tanis, Miranda Kusters, on behalf of the Dutch Lateral Node Imaging group

**Affiliations:** 1 Department of Surgery, Amsterdam UMC location Vrije Universiteit Amsterdam, De Boelelaan 1117, PO Box 7057, 1007 MB Amsterdam, the Netherlands; 2 Treatment and Quality of Life, Cancer Center Amsterdam, Amsterdam, the Netherlands; 3 Imaging and Biomarkers, Cancer Center Amsterdam, Amsterdam, the Netherlands; 4 Department of Radiology and Nuclear Medicine, Amsterdam UMC location Vrije Universiteit Amsterdam, De Boelelaan 1117, Amsterdam, the Netherlands; 5 Department of Radiology, The Netherlands Cancer Institute, Plesmanlaan 121, Amsterdam, the Netherlands; 6 GROW School for Oncology and Developmental Biology, University of Maastricht, Universiteitssingel 40, Maastricht, The Netherlands; 7 Department of Radiology, Department of Clinical Research, University of Southern Denmark, Odense University Hospital, Campusvej 55, DK-5230 Odense, Denmark; 8 Department of Surgery, Amsterdam UMC location University of Amsterdam, Meibergdreef 9, Amsterdam, the Netherlands; 9 Department of Surgical Oncology and Gastrointestinal Surgery, Erasmus MC, Doctor Molewaterplein 40, Rotterdam, the Netherlands

**Keywords:** Lateral lymph nodes, Rectal cancer, Magnetic resonance imaging

## Abstract

**Objectives:**

In patients with rectal cancer, the size and location of lateral lymph nodes (LLNs) are correlated to increased lateral local recurrence rates. Sufficient knowledge and accuracy when measuring these features are therefore essential. The objective of this study was to evaluate the variation in measurements and anatomical classifications of LLNs before and after training.

**Methods:**

Fifty-three Dutch radiologists examined three rectal MRI scans and completed a questionnaire. Presence, location, size, and suspiciousness of LLNs were reported. This assessment was repeated after a 2-hour online training by the same radiologists with the same three cases plus three additional cases. Three expert radiologists independently evaluated these 6 cases and served as the standard of reference.

**Results:**

Correct identification of the anatomical location improved in case 1 (62 to 77% (*p* = .077)) and in case 2 (46 to 72% (*p* = .007)) but decreased in case 3 (92 to 74%, *p* = .453). Compared to the first three cases, cases 4, 5, and 6 all had a higher initial consensus of 73%, 79%, and 85%, respectively. The mean absolute deviation of the short-axis measurements in cases 1–3 were closer—though not significantly—to the expert reference value after training with reduced ranges and standard deviations. Subjective determination of malignancy had a high consensus rate between participants and experts.

**Conclusion:**

Though finding a high consensus rate for determining malignancy of LLNs, variation in short-axis measurements and anatomical location classifications were present and improved after training. Adequate training would support the challenges involved in evaluating LLNs appropriately.

**Key Points:**

*• Variation was present in the assessment of the anatomical location and short-axis size of lateral lymph nodes.*

*• In certain cases, the accuracy of short-axis measurements and anatomical location, when compared to an expert reference value, improved after a training session.*

*• Consensus before and after training on whether an LLN was subjectively considered to be suspicious for malignancy was high.*

**Supplementary Information:**

The online version contains supplementary material available at 10.1007/s00330-022-08968-0.

## Introduction

Low, locally advanced rectal carcinomas (LARCs) have an increased chance of spreading towards lateral lymph nodes (LLNs), located in the lateral compartments [1]. These lateral compartments encompass the lymphatic tissue situated laterally of the mesorectal fascia and are not removed during standard total mesorectal excision (TME) surgery [2, 3]. While overall LR rates have decreased since the introduction of standardized neoadjuvant treatment and TME surgery, the proportion of lateral local recurrences (LLR) is increasing and currently accounts for approximately 50% of LRs [4]. This increase is most likely due to the still insufficient treatment of malignant LLNs.

Almost all nodal imaging studies have focused on mesorectal lymph nodes. Considering only the size of mesorectal nodes results in a low sensitivity and specificity for predicting malignancy (55–75%), but this can be improved to 85–100% when also considering morphological criteria (border contour, shape, signal heterogeneity) [5–8]. Contrastingly, recent literature regarding LLNs indicates that size and anatomical location, but not morphological criteria, are essential criteria to assess malignancy [9, 10]. Several studies have shown that the presence of enlarged LLNs on primary imaging increases the LLR rate to around 40% after 5 years [11–15]. A recent international, retrospective study including the re-review of MR-images for 1216 patients found significantly higher LLR rates for LLNs with a primary short-axis diameter of ≥ 7 mm; 19.5% after 5 years compared to 4.9% for patients without enlarged LLNs [10]. Furthermore, size and anatomical location were vital when also considering the restaging MRI. LLNs which remained > 4 mm and were located in the internal iliac compartment had a 5-year LLR rate of 52.3%. Obturator LLNs which remained > 6 mm had a 5-year LLR rate of 17.8% [9]. Morphological features were not found to have any significant influence on the prediction of malignancy.

There is, however, continued discussion as to the significance of LLNs, especially in Western cultures [2, 16]. Eastern clinics have long considered LLNs to be clinically significant and perform lateral lymph node dissections (LLND) for patients with LARC. This operation can lead to significant peri- and postoperative morbidity due to the possible damage of multiple blood vessels and/or nerves. While not performing an LLND in ‘high-risk’ patients may cause high LR rates, as found by Ogura et al [9, 10], an inadequate patient selection caused by, for example, over-staging LLNs on imaging, may mean that some patients unnecessarily undergo an LLND. An appropriate balance between these two, led by a suitable indication of malignancy by radiologists and active discussion by multidisciplinary teams (MDT) may help select the appropriate ‘high-risk’ patients who can benefit from an LLND, while sparing those for whom it is unnecessary. LLNs are still sparingly investigated, resulting in limited knowledge and awareness [2, 16, 17]. Accurate diagnosis of malignant LLNs remains therefore a challenging subject, even though subsequent treatment decisions for these patients are primarily based on the assessment of preoperative imaging [9, 10, 16, 18, 19].

What the size and anatomical location is of an LLN and whether it is considered malignant are vital pieces of information that should be accurately investigated, mentioned in radiology reports and discussed during MDT meetings [20, 21]. This study investigated if inter-physician variation was present for the measurement of LLN short-axis size, anatomical location, and for the judgement of whether an LLN is suspicious. This study also considered whether dedicated training resulted in significant improvements in these aspects.

## Methods

Participants in this study included radiologists who were either collaborators in the national ‘Snapshot rectal cancer 2016 study’ or interested colleagues. The Snapshot study is a national cross-sectional retrospective study investigating all patients who underwent an operation for rectal cancer in 2016 and includes re-assessment of all MR-imaging after a dedicated online training. The methodology of a Snapshot study is described in detail elsewhere [22].

**Fig. 1:**
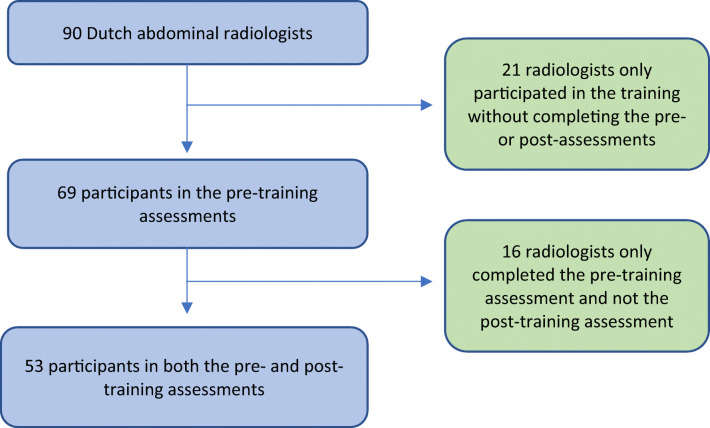
Flowchart of participants

A total of 90 Dutch abdominal radiologists from 62 hospitals participated in the online training session; 69/90 radiologists from 51/62 hospitals completed the necessary assessments before the training and 53/69 radiologists from 51/62 hospitals also completed the repeat assessments after the training session (Fig. 1). This latter group fulfilled the inclusion criteria for participation in the current study.

### Pre-training assessment

**Fig. 2:**
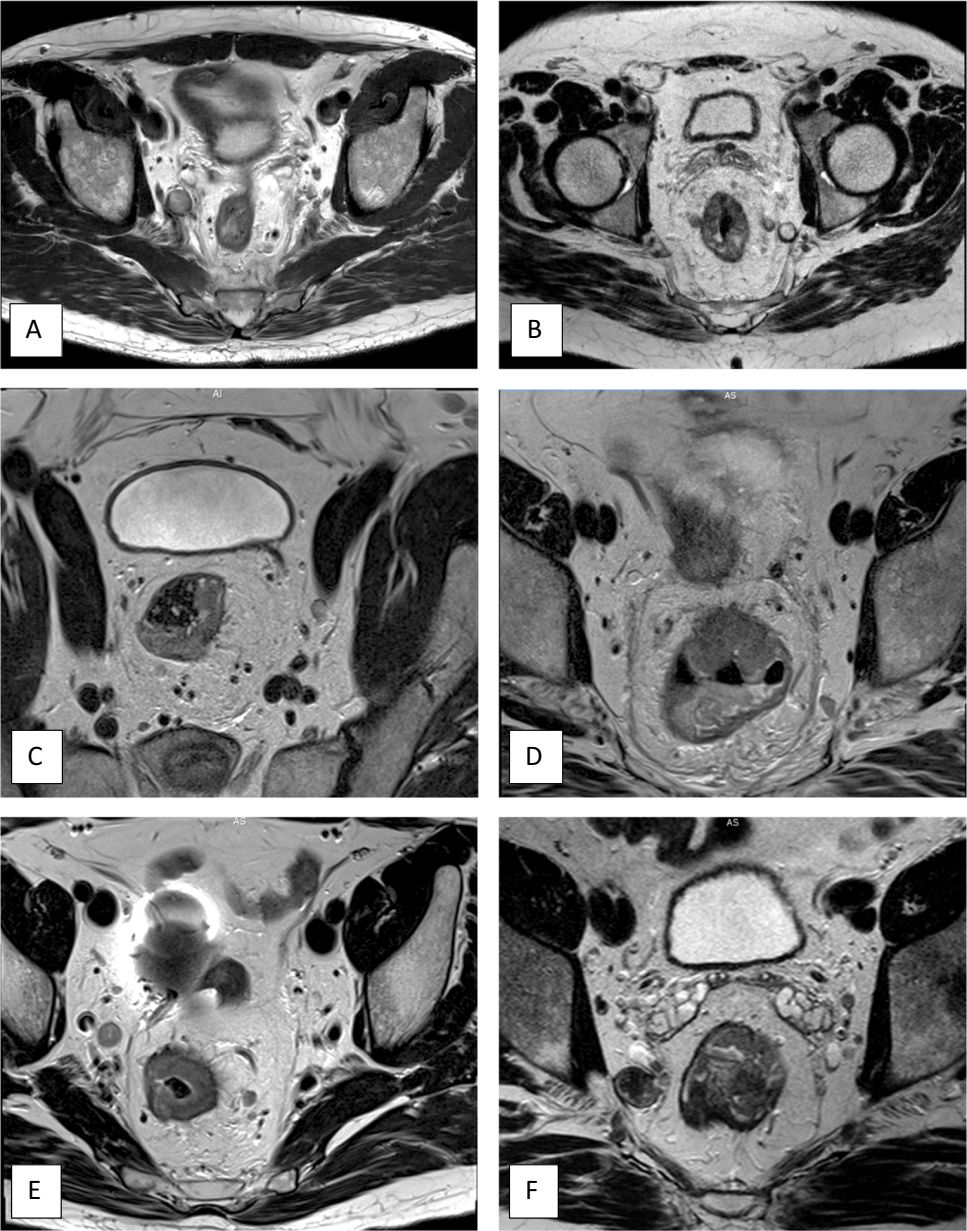
**A–F** Six cases with enlarged lateral lymph nodes used for the training. **A.** Right internal iliac compartment, expert reference measurement: 12.1 mm (11.6–12.5, SD 0.5 mm). **B.** Left obturator compartment, expert reference measurement: 12.1 mm (11.3–12.5, SD 0.6 mm). **C.** Left external iliac compartment, expert reference measurement: 7.2 mm (6.9–7.4, SD 0.2 mm). **D.** Left obturator compartment, expert reference measurement: 5.2 mm (4.8–5.9, SD 0.6 mm). **E.** Right internal iliac compartment, expert reference measurement: 9.1 mm (8.5–9.9, SD 0.7 mm). **F.** Right obturator compartment, expert reference measurement: 14.8 mm (14.3–15.4, SD 0.6 mm)

Participants were asked to complete a short questionnaire (Appendix 1) and then examine the primary MR-images of three cases with visible LLNs. All series of MRIs were provided. Cases 1 and 2 had one significantly enlarged LLN (internal iliac right and obturator left respectively), while case 3 had one LLN located in the external iliac compartment. Radiologists were asked to indicate the presence and number of LLNs, to measure the short-axis diameter of each LLN in mm, and to classify the anatomical compartment (Fig. 2a–f). Whether they deemed this LLN to be malignant was also reported.

### Training

**Fig. 3:**
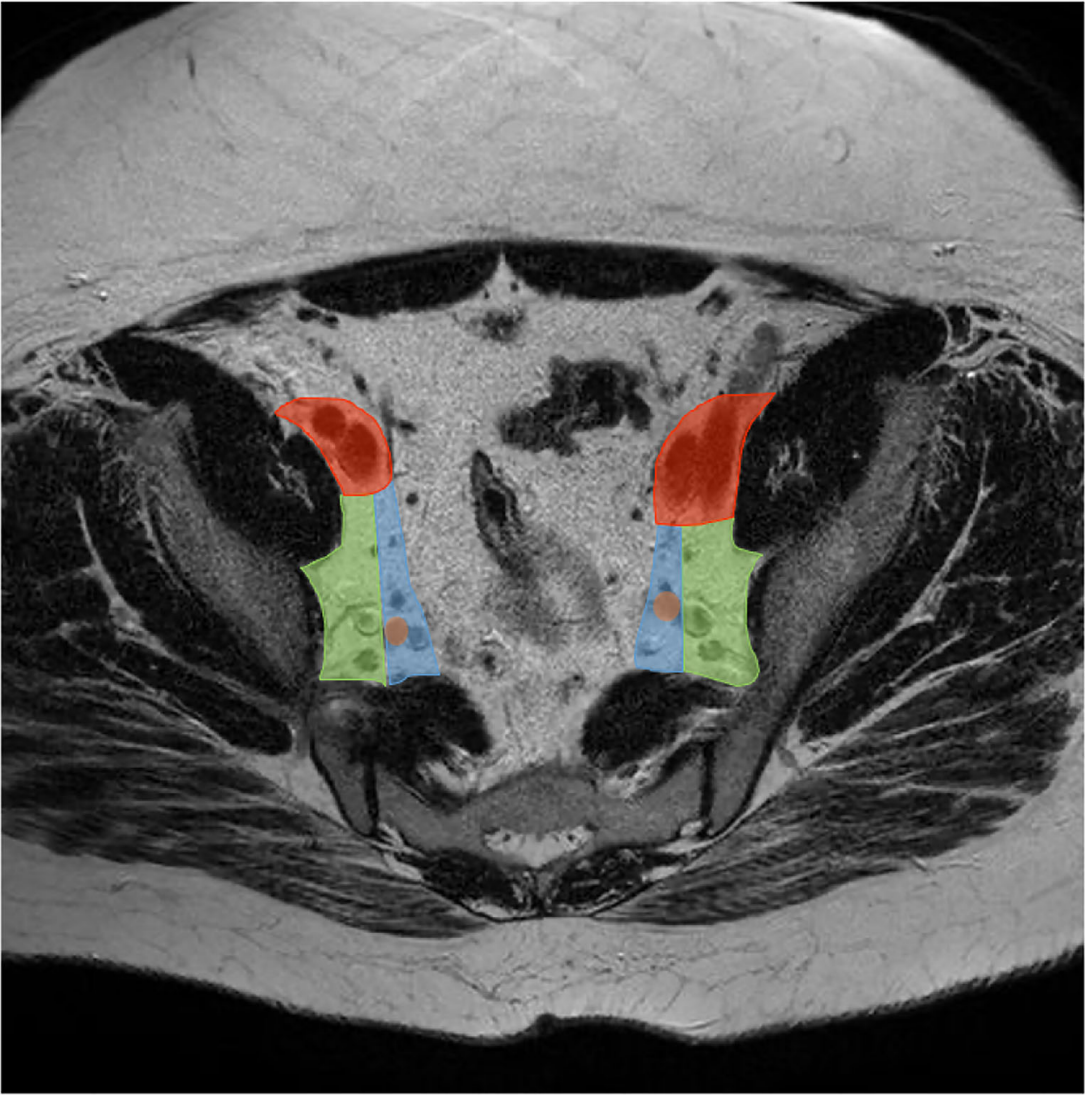
Axial view of T2-MRI depicting the lateral compartments. Red—external iliac compartment, green - obturator compartment, blue—internal iliac compartment. Brown spot: primary trunk of the internal iliac artery. The internal iliac compartment is defined as medial to the lateral border of the primary trunk of the internal iliac artery. The obturator compartment is the tissue located laterally of the primary trunk of the internal iliac artery and all tissue still present after the internal iliac artery exits the pelvis

Participants then partook in a 2-hour, interactive training led by two expert radiologists (K.H., R.B.T.) with 17 and 24 years’ experience, respectively. During the training, relevant background literature concerning LLNs was presented and 10 example MRI scans were shown, with particular emphasis on explaining how to discern between the different lateral compartments. Definitions of the anatomical compartments were described in accordance with previous literature [9, 18, 23]. An example is shown in Fig. 3.

### Post-training assessment

After the training, 53 participants scored the first three cases again, plus an additional three cases. Case 4 had multiple small LLNs (obturator left), while cases 5 (internal iliac right) and 6 (obturator right) had one significantly enlarged LLN (Fig. 2a–f). These three additional cases were included to evaluate the assessment in ‘new’ cases and also because one of the three primary cases (case 2) was shown and discussed during the training session.

### Standard of reference: expert evaluation

Three expert radiologists (R.B.T., D.L., K.H.) in the field of rectal cancer imaging independently evaluated the same six cases. The average of their measurements was used as the ‘expert reference value’ (ERV).

### Statistics

Data was analyzed in SPSS Statistics (version 26.0). Categorical data are described in *n* and percentages, continuous data as means and standard deviation (SD) or medians and inter-quartile range (IQR). The primary outcomes were the agreement with the expert reference regarding the short-axis diameter (in mm), location of LLNs, and suspiciousness. This was examined with either the McNemar change test or a single/paired t-test, depending on the type of data. Paired t-tests considered the mean absolute deviation (MAD) of the measurements from cases 1, 2, and 3, and these were compared to the ERV. A *p* value of < 0.05 was considered statistically significant.

## Results

Baseline characteristics of the 53 abdominal radiologists who participated in the study can be found in Table 1. Participants had a mean experience of 9.6 years (SD 5.4).

**Table 1:** Baseline characteristics

*N* = 53	
Years of experience, mean (SD)	9.6 (5.4)
Initial questionnaire:	
Present during multidisciplinary meetings?	33% (10–80%, SD 20)
What is your definition of a suspicious LLN?	30% ≥ 7 mm 43% same as mesorectal 27% other
How often do you report LLNs in your reports?	4% never 25% not often 36% sometimes 13% often 22% always
Are LLNs a sign of systemic disease?	54% no 21% neutral 25% yes
In what percentage of cT3/4 rectal cancer patients are LLNs present?	16% (5–70%, SD 9)

### Questionnaire

Participants first completed a questionnaire regarding LLNs. Participants estimated that LLNs were present in 16% (range 5–70%, SD 9) of patients with low, cT3/4 rectal cancer and various definitions were used to describe suspicious LLNs. A total of 30% used ≥ 7 mm as proposed by the Lateral Node Consortium Study [14, 15], 43% used the same criteria as for mesorectal lymph nodes and 27% gave different definitions such as 8mm, 10mm, or only based solely on the anatomical compartments. Only 22% of radiologists always mentioned LLNs in their reports. Finally, when asked if suspicious LLNs were a sign of metastatic disease, 54% replied no, 25% yes, and 21% remained neutral. Further results from the questionnaire can be found in Table 1.

### Short-axis measurements

**Fig. 4:**
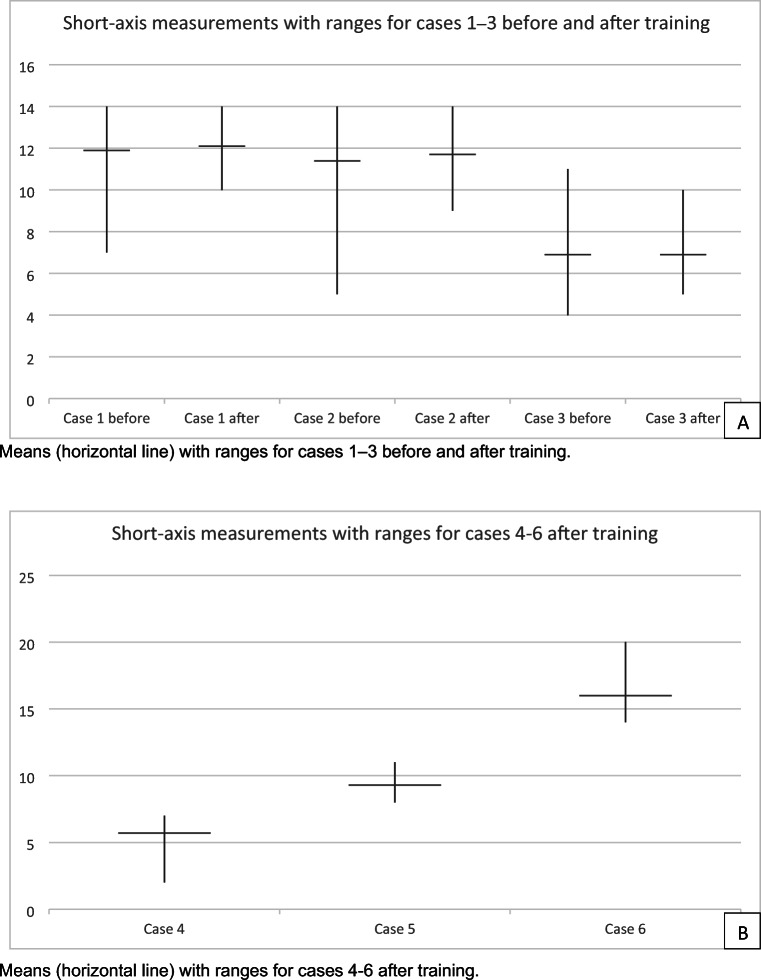
**A**, **B** Short-axis measurements for the 6 cases before and after training of the 53 participants. Means (horizontal line) with ranges for cases 1–3 before and after training (**A**). Means (horizontal line) with ranges for cases 4–6 after training (**B**)

Short-axis measurements of LLNs by participants before and after training compared to the expert reference value (ERV) are provided in Table 2. For cases 1, 2, and 3, no significant differences were observed in short-axis diameters or mean absolute deviation (MAD) by the participants compared to the expert reference before and after the training (*p* = 0.134–0.925). Reductions in MAD from the ERV, ranges, and SD were seen for all cases after training compared to before training (Fig. 4). Cases 4, 5, and 6 were only measured after the training (Table 2). Single t-tests compared to the ERV revealed significant differences for case 4 (ERV 5.2 (*p* = .003)) and case 6 (ERV 14.8 (*p* < .001)) but not for case 5 (ERV 9.1 (*p* = .079)).

**Table 2:** Cases 1–3 before and after training versus expert reference value

	Before training	After training	Expert reference
Case 1			
Short-axis measurements	11.9 mm (7.0–14.0, SD 1.1)	12.1 mm (10.0–14.0, SD 0.7)	12.1 mm (11.6–12.5, SD 0.4)
Compartment classification	62% right internal iliac vs. 38% right obturator	77% right internal iliac vs. 23% right obturator	Internal iliac (right)
Suspicious for malignancy	100% deemed LLN as suspicious on primary and restaging images	100% deemed LLN as suspicious on primary and restaging images	Deemed as suspicious by all three experts
Case 2: largest LLN			
Short-axis measurements	11.4 mm (5.0–14.0, SD 1.5)	11.7 mm (9.0–14.0, SD 1.0)	12.1 mm (11.3–12.5, SD 0.6)
Compartment classification	46% left obturator vs. 54% left internal iliac	72% left obturator vs. 28% left internal iliac	Obturator (left)
Suspicious for malignancy	100% deemed LLN as suspicious on primary and restaging images	98.1% deemed LLN as suspicious on primary and restaging images	Deemed as suspicious by all three experts
Case 3			
Short-axis measurements	6.9 mm (4.0–11.0, SD 1.7)	6.9 mm (5.0–10.0, SD 1.6)	7.2 mm (6.9–7.4, SD 0.2)
Compartment classification	92% left external iliac, 2.7% left internal iliac, 5.3% left obturator	74% left external iliac, 17.2% left obturator, 8.8% left internal iliac	External iliac (left)
Suspicious for malignancy	21% deemed LLN as suspicious on primary images	15% deemed LLN as suspicious on primary images	Not deemed as suspicious by all three experts
Case 4			
Short-axis measurements	Not applicable	5.7 mm (2.0–7.0, SD 1.0)	5.3 mm (4.8–5.9, SD 0.5)
Compartment classification	Not applicable	79% left obturator vs. 21% left internal iliac	Obturator (left)
Suspicious for malignancy	Not applicable	23.7% deemed LLN as suspicious on primary images	Not deemed as suspicious by all three experts
Case 5			
Short-axis measurements	Not applicable	9.3 mm (8.0–11.0, SD 0.7)	9.1 mm (8.6–9.9, SD 0.7)
Compartment classification	Not applicable	76% right internal iliac vs. 24% right obturator	Internal iliac (right)
Suspicious for malignancy	Not applicable	100% deemed LLN suspicious on primary images and 23.1% still suspicious on restaging images	Deemed as suspicious by all three experts, but responded well to neoadjuvant treatment
Case 6			
Short-axis measurements	Not applicable	16.0 mm (14.0–20.0, SD 1.4)	14.8 mm (14.3–15.4, SD 0.5)
Compartment classification	Not applicable	85% right obturator vs. 15% right internal iliac	Obturator (right)
Suspicious for malignancy	Not applicable	100% deemed LLN suspicious on primary images and 98.1% still suspicious on restaging images	Deemed as suspicious by all three experts

Variations and standard deviations in measurements between participants decreased after training, and smaller ranges and standard deviations were seen between the three expert radiologists compared to the participants (Appendix 1).

### Location of LLNs according to anatomical compartments

The three experts reached full agreement on the anatomical locations of the LLNs in all six cases (Table 2 and Appendix 1). Consensus regarding the location of the largest LLN in case 1 was initially 62% (right internal iliac) and increased to 78% after training (*p* = .077). Consensus for the location of the largest LLN in case 2 was initially 46% (left obturator) and increased to 72% after training (*p* = .007). The external iliac LLN in case 3 had an initial consensus of 92%, but this decreased to 74% after training (*p* = .453). Consensus for the anatomical locations of cases 4, 5, and 6 was 74, 79%, and 85%, respectively (similar to the post-training results of cases 1–3) and all were in line with the expert reference.

### Malignant LLNs

There was a high consensus between the participating radiologists and experts regarding whether an LLN was considered suspicious. The three experts stated independently from each other that cases 1, 2, and 6 were suspicious on both the primary and restaging images. Case 5 was suspicious primarily, but responded well to neoadjuvant treatment, while cases 3 and 4 were not deemed as suspicious for malignancy. For cases 1, 2, and 6, the consensus between participants was 98.1–100% that the LLNs here were suspicious, while 15–23.7% also considered LLNs in cases 3 and 4 to be suspicious. All participants regarded the LLN in case 5 as initially suspicious (100%), and 23.1% also stated its suspiciousness when considering restaging images (Table 2).

## Discussion

This study, including 53 Dutch abdominal radiologists from 51 hospitals, demonstrates that inter-physician variation is present for the short-axis measurements of LLNs and for classifying the appropriate lateral compartment. This variation decreased when repeating the assessment after completing a 2-hour training provided by expert radiologists. Contrastingly, the overall consensus between participants and compared to the experts was very high for the subjective determination of whether an LLN was suspicious of malignancy.

Participants indicated prior to the training that they used different definitions to diagnose suspicious LLNs in their clinical practice (Table 1) and there was a wide range in whether the participants routinely reported the presence or absence of LLNs in their reports; 3% said never and 22% always. It is possible that radiologists only mention their presence if deemed suspicious, even though heterogeneity existed within the group regarding their definitions for ‘suspiciousness’. This reflects variation between participating hospitals and exposes a lack of knowledge and awareness for LLNs by a proportion of participants. Another difference was the interpretation of whether LLNs are indicative of metastatic or locoregional disease; 23% believed them to represent metastatic disease, 56% locoregional and 21% indicated that they did not know how to answer this question. Recent literature has suggested that LLNs represent locoregional disease, considering that distant metastasis rates appear not to be influenced by the presence of enlarged LLNs [2, 9, 10].

**Fig. 5:**
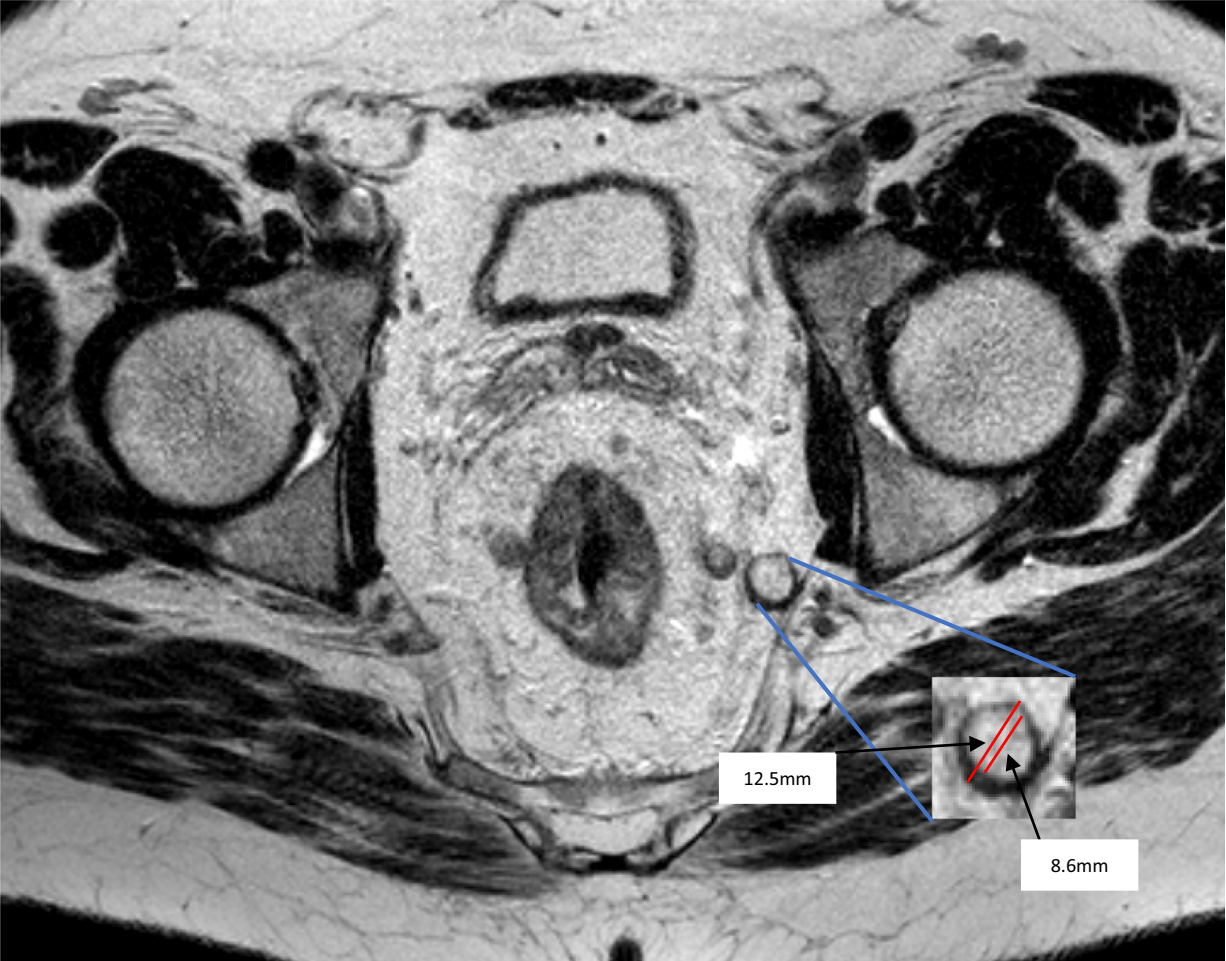
Variation between two potential measurements of the largest LLN from case 2. Axial T2-MRI with a zoomed-in image of the largest LLN from case 2. Two measurements display the variation possible for this LLN due to the presence on a thick outer shell

Since research has found that size, and not morphological features, is primarily relevant to LLNs [9, 10]; it is vital that size measurements are reported accurately. Based on the most recent retrospective research, 7 mm (short-axis) has been suggested as the threshold for malignancy [9, 10]. One issue with selecting a specific size threshold is the inter-physician variation in measurements found in this study. While a small range of variation is to be expected when comparing measurements of multiple specialists, the ranges found here were larger than expected. For example, in case 2, while the mean measurement was 11.4 mm, and thus close to the ERV of 12.1 mm, measured values between participants ranged from 5 to 14 mm (SD 1.5). This range significantly decreased after the training to 9–14 mm (SD 1.0). This broad variation may be explained by a significant outer ‘shell’ illustrated in Fig. 5. In fact, for all cases, the ranges and standard deviations in measurements decreased after the training. This is most likely a direct result of the training, during which dedicated time was spent explaining short-axis measurements with MRI examples.

An important consideration should be the variation in measurements for the LLNs which straddle the 7 mm size. For these LLNs, the chances of discrepant measurements and opinions on suspiciousness increased, compared to larger LLNs for which consensus in this study was almost 100% that it was suspicious. This variation was also visible in the range of measurements between the three experts for LLNs around 7 mm in size (Appendix 1). For both the participants and experts, it is possible that the measurements from one individual vary just below or just above the 7 mm threshold compared to a colleague; insinuating that such a strict guideline may be subject to inter-physician variation. In such cases, peer review by a colleague could be desirable and included in protocols [24, 25]. 

This study also found that initial consensus regarding the anatomical location of LLNs in the lateral compartments was low; suggesting that various borders are currently used to define the compartments. During the training session, the anatomical definitions were described according to Ogura et al [9] and Gollub et al [23] and were explained with the help of multiple visual examples. For the cases completed after the training, a noticeable improvement was seen in the consensus regarding anatomical location. This implies that these borders are understandable and translatable into routine daily practice. However, correct identification of the compartments was still not consistently > 80% after the training, with the highest consensus of 86% for case 6. Considering the oncological implications for LLNs in different locations [18, 26], it is important that LLNs are classified correctly. Possibilities could include color atlases with detailed explanations combined with interactive training sessions, as this does appear to be advantageous. On the other hand, while cases 1 and 2 improved after training, case 3 consensus worsened. This is due to the fact that during the training session, experts explained that elongated LLNs located close to the external iliac artery were rarely found to be malignant. Consequently, less radiologists mentioned the presence of the node in case 3 because they did not consider it to be a *lateral* lymph node anymore.

Interestingly, high consensus was seen between participants when asked whether an LLN was considered malignant, demonstrating a good ability to judge this phenomenon. Mentioning this in reports is also essential so to influence potential treatment decisions by radiation oncologists and surgeons. Results from the questionnaire found that only 22% always reported LLNs, a rate that must be improved. Methods to facilitate this could be structured reporting, focused training, or computer-aided detection to ensure that suspicious LLNs are properly identified as signaled to the multidisciplinary team [27]. A clear international guideline and/or universal definition for when an LLN is considered malignant is currently missing. Considering the multidisciplinary treatment implications of LLNs discussed here, concise guidelines should be created and implemented.

This study has a number of limitations. Firstly, not all participants who completed the pre-training assessment also participated in the post-training assessment, reflecting a selection bias for the 53 participants, possibly representing the more enthusiastic participants. The fact that the total case number before training was only three is a limiting factor, restricting our ability to properly evaluate their ability and knowledge. Additionally, while the addition of three new cases afterwards was carefully considered to allow an analysis of the ‘new’ consensus after training, this method creates heterogeneity within the small sample of cases. Furthermore, the ERV is based on the measurements of three experts for which variation is also possible. Lastly, many of the hospitals had one representative radiologist, while intra-institution variation would also be very interesting to investigate.

## Conclusion

This study found that while radiologists have a high consensus rate for the subjective determination of malignancy of lateral lymph nodes (LLNs), variation was present in the classification of LLNs into lateral compartments and their short-axis measurements. These aspects improved after participating in an online training session led by expert radiologists. Considering the oncological implications that LLNs hold; especially with regard to the differences found between lateral compartments and for varying short-axis sizes, care should be taken to ensure a minimal amount of variation between specialists. Increased knowledge and awareness through training, instruction documents, and visual guides may help improve this further.

## Supplementary information

ESM 1 (DOCX 19 kb)

## Abbreviations

ERV: Expert reference value
LLND: Lateral lymph node dissection
LLNs: Lateral lymph nodes
LLR: Lateral local recurrence
LR: Local recurrence
MAD: Mean absolute deviation
MRI: Magnetic resonance imaging
TME: Total mesorectal excision

## Declarations

### Guarantor

The scientific guarantor of this publication is Dr. Miranda Kusters.

### Statistics and biometry

No complex statistical methods were necessary for this paper.

### Methodology

• Prospective

• observational

• multicentre study

## References

[1] Steup WH, Moriya Y, van de Velde CJ (2002). Patterns of lymphatic spread in rectal cancer. A topographical analysis on lymph node metastases. Eur J Cancer.

[2] Christou N, Meyer J, Toso C, Ris F, Buchs NC (2019). Lateral lymph node dissection for low rectal cancer: Is it necessary?. World J Gastroenterol.

[3] Sammour T, Chang GJ (2018). Lateral pelvic lymph node dissection and radiation treatment for rectal cancer: Mutually exclusive or mutually beneficial?. Ann Gastroenterol Surg.

[4] Iversen H, Martling A, Johansson H, Nilsson PJ, Holm T (2018). Pelvic local recurrence from colorectal cancer: surgical challenge with changing preconditions. Color Dis.

[5] Kim JH, Beets GL, Kim MJ, Kessels AG, Beets-Tan RG (2004). High-resolution MR imaging for nodal staging in rectal cancer: are there any criteria in addition to the size?. Eur J Radiol.

[6] Lahaye MJ, Engelen SM, Nelemans PJ (2005). Imaging for predicting the risk factors--the circumferential resection margin and nodal disease--of local recurrence in rectal cancer: a meta-analysis. Semin Ultrasound CT MR.

[7] Brown G, Richards CJ, Bourne MW (2003). Morphologic predictors of lymph node status in rectal cancer with use of high-spatial-resolution MR imaging with histopathologic comparison. Radiology.

[8] Bipat S, Glas AS, Slors FJ, Zwinderman AH, Bossuyt PM, Stoker J (2004). Rectal cancer: local staging and assessment of lymph node involvement with endoluminal US, CT, and MR imaging--a meta-analysis. Radiology.

[9] Ogura A, Konishi T, Beets GL (2019). Lateral nodal features on restaging magnetic resonance imaging associated with lateral local recurrence in low rectal cancer after neoadjuvant chemoradiotherapy or radiotherapy. JAMA Surg.

[10] Ogura A, Konishi T, Cunningham C (2019). Neoadjuvant (chemo)radiotherapy with total mesorectal excision only is not sufficient to prevent lateral local recurrence in enlarged nodes: results of the multicenter lateral node study of patients with low cT3/4 rectal cancer. J Clin Oncol.

[11] Ueno H, Mochizuki H, Hashiguchi Y, Hase K (2001). Prognostic determinants of patients with lateral nodal involvement by rectal cancer. Ann Surg.

[12] Ueno M, Oya M, Azekura K, Yamaguchi T, Muto T (2005). Incidence and prognostic significance of lateral lymph node metastasis in patients with advanced low rectal cancer. Br J Surg.

[13] Watanabe T, Muro K, Ajioka Y (2018). Japanese Society for Cancer of the Colon and Rectum (JSCCR) guidelines 2016 for the treatment of colorectal cancer. Int J Clin Oncol.

[14] Kim MJ, Hur BY, Lee ES (2018). Prediction of lateral pelvic lymph node metastasis in patients with locally advanced rectal cancer with preoperative chemoradiotherapy: focus on MR imaging findings. PLoS One.

[15] Kim MJ, Kim TH, Kim DY (2015). Can chemoradiation allow for omission of lateral pelvic node dissection for locally advanced rectal cancer?. J Surg Oncol.

[16] Williamson JS, Quyn AJ, Sagar PM (2020). Rectal cancer lateral pelvic sidewall lymph nodes: a review of controversies and management. Br J Surg.

[17] Kim MJ, Oh JH (2018). Lateral lymph node dissection with the focus on indications, functional outcomes, and minimally invasive surgery. Ann Coloproctol.

[18] Schaap DP, Boogerd LSF, Konishi T (2021). Rectal cancer lateral lymph nodes: multicentre study of the impact of obturator and internal iliac nodes on oncological outcomes. Br J Surg.

[19] Schaap DP, Ogura A, Nederend J (2018). Prognostic implications of MRI-detected lateral nodal disease and extramural vascular invasion in rectal cancer. Br J Surg.

[20] Hazen SJA, Sluckin TC, Konishi T, Kusters M (2021) Lateral lymph node dissection in rectal cancer: State of the art review. Eur J Surg Oncol. 10.1016/j.ejso.2021.11.003 10.1016/j.ejso.2021.11.003 34802862

[21] Sluckin TC, Hazen SJA, Kusters M (2021). From “East vs West” towards international multidisciplinary collaboration: an appraisal of lateral lymph nodes in rectal cancer. Ann Gastroenterol Surg.

[22] Dutch Snapshot Research Group (2017). Benchmarking recent national practice in rectal cancer treatment with landmark randomized controlled trials. Color Dis.

[23] Gollub MJ, Lall C, Lalwani N, Rosenthal MH (2019). Current controversy, confusion, and imprecision in the use and interpretation of rectal MRI. Abdom Radiol (NY).

[24] Chaudhry H, Del Gaizo AJ, Frigini LA et al (2020) Forty-one million RADPEER reviews later: what we have learned and are still learning. J Am Coll Radiol 17(6):779–785 10.1016/j.jacr.2019.12.023 31991118

[25] Chetlen AL, Petscavage-Thomas J, Cherian RA (2020). Collaborative learning in radiology: from peer review to peer learning and peer coaching. Acad Radiol.

[26] Akiyoshi T, Watanabe T, Miyata S, Kotake K, Muto T, Sugihara K (2012). Results of a Japanese nationwide multi-institutional study on lateral pelvic lymph node metastasis in low rectal cancer: is it regional or distant disease?. Ann Surg.

[27] Bruno MA, Walker EA, Abujudeh HH (2015). Understanding and Confronting Our Mistakes: The Epidemiology of Error in Radiology and Strategies for Error Reduction. Radiographics.

