# Supplementary material for: Significant improvement after training in the assessment of lateral compartments and short-axis measurements of lateral lymph nodes in rectal cancer
Source: Eur Radiol. 2022 Jul 8;33(1):483–92. doi: 10.1007/s00330-022-08968-0 (PMC9755077; doi:10.1007/s00330-022-08968-0)
Supplement: Supplementary file 1 — (DOCX 19 kb) [file 330_2022_8968_MOESM1_ESM.docx]

Appendix 1: Short-axis measurements by three expert radiologists for the creation of expert reference value (ERV)

| ***Short-axis measurements*** | **Expert 1** | **Expert 2** | **Expert 3** | **Mean, range (SD)** |
| --- | --- | --- | --- | --- |
| **Case 1** | 12.5mm | 11.6mm | 12.1mm | 12.1, 11.6-12.5 (0.5) |
| **Case 2** | 12.5mm | 12.4mm | 11.3mm | 12.1, 11.3-12.5 (0.6) |
| **Case 3** | 7.4mm | 7.3mm | 6.9mm | 7.2, 6.9-7.4 (0.2) |
| **Case 4** | 5.9mm | 5.0mm | 4.8mm | 5.2, 4.8-5.9 (0.6) |
| **Case 5** | 9.9mm | 8.8mm | 8.6mm | 9.1, 8.6-9.9 (0.7) |
| **Case 6** | 15.4mm | 14.3mm | 14.8mm | 14.8, 14.3-15.4 (0.6) |

Appendix 2: Questionnaire

**Questionnaire lateral lymph nodes**

**Name & date**:

**Hospital:**

**Position:**

**Years of experience:**

1. How often are you present at MDT meetings in which patients with colorectal cancer are discussed? Please answer in percentages.
2. How many patients with a low, cT3/4 rectal carcinoma do you think have suspicious lateral lymph nodes on the primary MRI? Please answer in percentages.
3. What do you use as a definition for a suspicious lateral lymph node?
4. Do you think that there is a clear definition used in the Netherlands to describe/determine suspicious lateral lymph nodes?

**Completely Completely
disagree agree**

1. How often do you report the presence of lateral lymph nodes in your primary MRI report for patients with low, cT3/4 rectal carcinoma, even if they are not suspicious?

**Never Always**

1. How much do you agree with the following statement: “When suspicious lateral lymph nodes are present on the primary MRI without distant metastases, then this is classified as systemic disease.”

**Completely Completely
disagree agree**
